# Unmasking Sturge–Weber syndrome in adulthood: a case with extrafacial port-wine stain and delayed neurological symptoms

**DOI:** 10.1097/MS9.0000000000002049

**Published:** 2024-04-15

**Authors:** Pramodman Singh Yadav, Pratik Adhikari, Binod Mehta, Shuvam Khadka, Min Raj Bhurtel, Alok Dahal, Sujan Acharrya

**Affiliations:** B.P. Koirala Institute of Health Sciences, Dharan, Nepal

**Keywords:** leptomeningeal angiomatosis, neurocutaneous disorder, port-wine stain, Sturge–Weber syndrome

## Abstract

**Background:**

Sturge–Weber syndrome (SWS) is a rare neurocutaneous disorder characterized by a facial port-wine birthmark, leptomeningeal angiomatosis, and glaucoma. This case report highlights the challenges of diagnosing SWS when presenting with atypical features. Here, the authors present a 55-year-old man with an extrafacial port-wine stain and delayed-onset seizures, deviating from the classic triad.

**Case presentation:**

A 55-year-old man presented with a recent seizure and a characteristic port-wine birthmark extending beyond the typical facial region. Neurological examination revealed no weakness, speech difficulties, or coordination problems. Ophthalmological examination didn’t reveal glaucoma. Limited resources restricted access to advanced imaging like MRI scans. However, based on the constellation of clinical findings, including the facial birthmark with angiomatosis and the new-onset seizure, the patient received a diagnosis of SWS. Treatment with Levetiracetam was initiated to prevent future seizures, and patient education on managing diabetes and hypertension was provided.

**Clinical discussion:**

This case underscores the importance of considering SWS in diagnosing adult-onset seizures, especially with a characteristic facial birthmark. The delayed presentation and isolated seizure suggest potentially less severe brain involvement. Resource limitations necessitated a clinical diagnosis and treatment with readily available medications.

**Conclusion:**

This case highlights the challenges of diagnosing atypical SWS presentations. Early diagnosis is crucial for prompt management and improved patient outcomes. Future research should focus on developing robust diagnostic tools and exploring novel treatment options for atypical SWS presentations.

## Background

Highlights

- This case report highlights Sturge–Weber syndrome (SWS), a rare neurocutaneous disorder, and its diverse presentations across different age groups.
- A 55-year-old man with diabetes and high blood pressure presented with a seizure episode and distinctive purple facial spots, leading to the diagnosis of SWS characterized by a port-wine stain.
- SWS can manifest with seizures, visual impairments, and neurological deficits. Diagnosis involves brain imaging to confirm leptomeningeal angiomas and calcifications.
- Tailored interventions, such as antiepileptic treatment and laser therapy for port-wine stains, are essential for managing SWS and improving patient outcomes.

Sturge–Weber syndrome (SWS), also known as encephalotrigeminal angiomatosis, is a rare neurocutaneous condition. It distinguishes itself through a single facial naevus flammeus (also known as a port-wine stain) and ipsilateral leptomeningeal angiomatosis. Schirmer was the first to describe it in 1860, and Sturge was more detailed in 1879. Leptomeningofacial angiomatosis, Sturge–Weber illness, and Sturge–Weber-Dimitri syndrome are other names for it^1,2^. It is characterized by a classic triad:

- Facial naevus flammeus (port-wine stain): A unilateral, reddish-purple birthmark typically following the ophthalmic (V1) and maxillary (V2) divisions of the trigeminal nerve on the forehead and upper eyelid^1,2^.
- Ipsilateral leptomeningeal angiomatosis: Slow-growing vascular malformations involving the leptomeninges on the same side of the body as the facial birthmark^2^.
- Glaucoma (variable): Increased intraocular pressure due to abnormal blood vessel development in the eye^1^.

SWS arises from a post-zygotic mutation in the GNAQ gene, leading to uncontrolled endothelial cell proliferation and vascular malformations^3^. These malformations can cause a spectrum of complications depending on their location and size. In the brain, leptomeningeal angiomatosis can lead to seizures, hemiparesis, intellectual disability, and visual field deficits, with severity often correlating to the extent of the malformation^2^.

Intraoral angiomatosis may affect the lips, leading to macrocheilia and hemihypertrophy of the buccal mucosa, palate, and oral floor. The degree of gingival involvement can range from mild vascular hyperplasia to severe overgrowth that makes closing the mouth difficult or nearly impossible^4^.

Roach divided SWS variants into three categories in 1992:

- Type I: The person has a port-wine stain on their face, a leptomeningeal angioma, and they may also have glaucoma.
- Type II: The person has a port-wine stain on their face, no leptomeningeal angioma, and they could have glaucoma.
- Type III: The person has glaucoma infrequently, no face port-wine stain, and leptomeningeal angiomatosis^5^.

Seizures of the atonic, tonic, or myoclonic variety, with onset ages ranging from infancy to 23 years, are the most prevalent neurologic manifestations of SWS that present for diagnosis^6^. It is estimated that this illness affects 1 in 20 000–50 000 live births, despite the fact that the incidence is not accurately reported^3^. SWS has no preference for any particular race or gender^3^.

SWS can present with atypical features, making diagnosis challenging. In this case study, we present a unique presentation of SWS in a 50-year-old man with a characteristic port-wine birthmark extending beyond the typical facial region (mention specific location if relevant). Notably, he presented with a recent seizure and altered sensorium, which are common neurological manifestations but can have a delayed onset.

## Case presentation

A 55-year-old man from a rural community presented to our clinic complaining of a recent episode (2 days ago) characterized by involuntary jerking movements of his entire body and a brief loss of consciousness. He reported his face turning a deep purple colour on the left side during the episode, but this discoloration faded within minutes. He denied any fever, headache, vomiting, or difficulty seeing or moving since the event.

His medical history was significant for poorly controlled diabetes and high blood pressure. He never had seizures as an adult or child and denied any family history of similar problems. He quit smoking and drinking alcohol 4 years ago.

### Physical examination

#### Neurological examination

Vital signs were within normal limits. The neurological examination revealed no weakness, speech difficulties, or problems with coordination. This suggests relatively preserved motor and cognitive function at the time of presentation.

#### Skin findings

**Figure 1:**
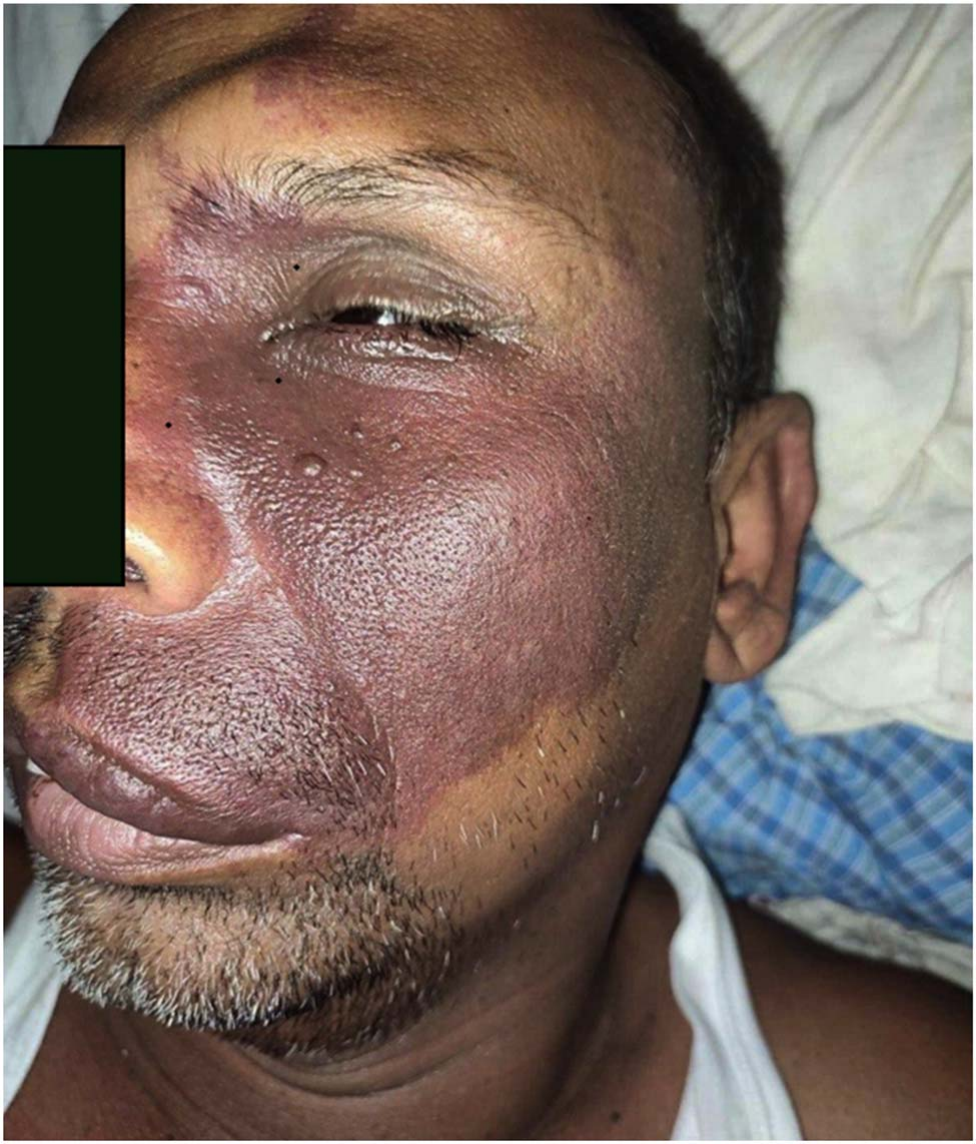
Purple lesions are present over the skin on the left side of the face in the frontal region, nose, eyelid, cheek, and lips, along with hypertrophy of soft tissue.

A striking feature was the presence of a large, port-wine-coloured birthmark on the left side of his face. The birthmark extended from his forehead across his nose, cheek, eyelid, and down to his lips (Fig. 1).

The affected area also exhibited a noticeable thickening and enlargement of the underlying soft tissue (angiomatosis). This constellation of findings (facial port-wine birthmark with angiomatosis) is highly suggestive of SWS.

#### Eye findings

An ophthalmologic examination using a handheld lens revealed no signs of glaucoma, a common complication of SWS. However, a more comprehensive eye examination with specialized equipment might be necessary to rule out other potential ocular involvement associated with SWS.

### Syndrome (SWS)

#### Brain findings

Unfortunately, access to advanced imaging techniques like MRI scans was limited in our setting. Therefore, we couldn’t directly visualize the brain to confirm potential abnormalities associated with SWS, such as calcifications or malformations.

#### Other systemic findings

Routine blood tests showed normal results, including complete blood count, electrolytes, and liver function tests (Table 1). A chest X-ray, ultrasonography (USG) of the abdomen and pelvis and X- ray of skull (Table 2) were unremarkable. No other significant findings were identified on examination.

**Table 1:** Routine blood investigations

Name of the investigations	Values
Haemoglobin (gm%)	12.7
Total count (per cumm)	10 300
Differential count (N/L/E/M %)	55/45/02/02
Platelet count (per cumm)	3 73 000
Random blood sugar (mg/dl)	95
Urea (mg/dl)	27
Creatinine (mg/dl)	0.89
Total bilirubin(mg/dl)	1.0
Direct bilirubin (mg/dl)	0.3
Indirect bilirubin (mg/dl)	0.7
Sodium (mmol/l)	140
Potassium (mmol/l)	4.5
Magnesium (mg/dl)	1.8
Ionized calcium (mmol)	1.4
Urine routine micro	Normal

N/L/E/M, neutrophil/lymphocyte/eosinophil/monocyte.

**Table 2:** Radiological investigations summary

Investigations	Reports
Ultrasonography of abdomen and pelvis	Normal
Skull X-ray	Normal
Chest X-ray	Normal

Based on the constellation of clinical findings, the patient was diagnosed with SWS. The presence of a new-onset seizure with a characteristic facial port-wine birthmark involving the left side of the face and angiomatosis strongly suggested SWS. While an EEG and MRI scan would have been ideal to confirm the diagnosis and assess brain involvement, the limited resources in our setting necessitated a clinical diagnosis based on the available information.

Given the limitations, we couldn’t perform a full workup or offer advanced treatment options. However, we initiated treatment with a readily available anti-seizure medication (Levetiracetam) to prevent future seizures. We also counselled the patient on the importance of managing his diabetes and hypertension, as these conditions can worsen neurological outcomes. Unfortunately, due to limited resources, we couldn’t offer him laser treatment for the facial birthmark. However, we provided him with educational materials about SWS and encouraged him to seek care at a more specialized facility if possible in the future.

This case highlights the challenges and considerations involved in diagnosing and managing SWS in resource-limited settings. The diagnosis primarily relied on the characteristic clinical features, and basic investigations like blood tests and chest X-rays provided additional support. Management strategies were adapted to available resources, and patient education with a potential referral for advanced evaluation was crucial.

I am writing in accordance with the SCARE checklist. In accordance with the SCARE 2023 guideline (Sohrabi *et al*., 2023), the methodology for reporting surgical case details was strictly adhered to in this study.^7^

## Discussion

Unlike most SWS presentations, where seizures and other neurological symptoms manifest in childhood^8^, this patient’s first seizure occurred at the relatively uncommon age of 55. Additionally, he only experienced a single seizure episode, whereas SWS patients often have recurrent seizures^9^.

The limited resources in our setting prevented access to advanced imaging like MRI scans. However, the characteristic facial port-wine birthmark with angiomatosis and the new-onset seizure were sufficient for a clinical diagnosis of SWS. This aligns with some studies exploring SWS diagnosis in resource-constrained environments^10,11^.

The patient’s relatively preserved cognitive function and lack of recurrent seizures suggest potentially less severe brain involvement. This could explain the delayed manifestation of neurological symptoms^9^. Alternatively, the leptomeningeal angiomas, a hallmark of SWS, can grow and change over time. While the facial birthmark was present since birth, the angioma in the brain may have progressed to a point where it triggered the seizure at a later age^12^.

This case underscores the importance of considering SWS in the differential diagnosis of adult-onset seizures, especially if accompanied by a characteristic facial port-wine birthmark^8^. It highlights the value of clinical acumen and the ability to utilize readily available investigations for diagnosis in resource-limited settings^10,11^.

The absence of advanced imaging like MRI scans limits a definitive confirmation of brain involvement. Further evaluation in a more specialized facility would be ideal for a comprehensive assessment. The patient should be encouraged to seek this opportunity, if possible, to explore the potential benefits of advanced diagnostics and treatment options like laser therapy for the facial birthmark^9^.

In summary, this case of SWS with a delayed presentation and isolated seizure emphasizes the need for considering SWS in adult-onset seizures with a characteristic facial birthmark^8^. It also highlights the importance of resource-adapted diagnostic approaches and the value of clinical expertise in such settings.

## Conclusions

In conclusion, this case report highlights the diagnostic challenges associated with atypical presentations of SWS. The patient’s extrafacial port-wine stain and delayed onset of seizures deviated from the classic triad, potentially leading to a delay in diagnosis. This case underscores the importance of maintaining a high index of suspicion for SWS in patients with any suggestive features, regardless of typical presentation. Early diagnosis allows for prompt management of neurological complications and can improve patient outcomes. Future research efforts should focus on developing more robust diagnostic tools and exploring novel treatment options for SWS patients with atypical presentations.

## Ethics approval and consent to participate

Not applicable.

## Consent for publication

Informed consent was taken from the patient to publish this case report.

## Sources of funding

No funding was obtained for this study.

## Author contribution

S.K. provided us with data and materials from the archive and their notes. P.S.Y., P.A., B.M., S.A. and M.R.B. wrote the manuscript, collected the images and put them in perspective according to the timeline of the case. A.D. reviewed the manuscript and did the final editing. All the authors read the final manuscript and approved the case.

## Conflicts of interest disclosure

The authors declare that they have no competing interests.

## Research registration unique identifying number (UIN)

This is a case report so registration was not required.

## Guarantor

Dr Pratik Adhikari is the guarantor of the study.

## Availability of data and materials

The datasets supporting the conclusions of this article are included within the article.

## Provenance and peer review

Not commissioned or externally peer-reviewed.
